# Immunoinfiltration Analysis of Mitochondrial Damage‐Related Genes in Lung Adenocarcinoma and Construction of a Classification and Prognostic Model Integrated With WGCNA and Machine Learning Algorithms

**DOI:** 10.1002/cam4.70590

**Published:** 2025-01-16

**Authors:** Jirong Zhang, Lin Lin

**Affiliations:** ^1^ Department of Geriatrics The Second Affiliated Hospital of Harbin Medical University Harbin Heilongjiang People's Republic of China; ^2^ Department of Respiratory Medicine The Second Affiliated Hospital of Harbin Medical University Harbin Heilongjiang People's Republic of China

**Keywords:** diagnostic model, lung adenocarcinoma, machine learning, prognostic model

## Abstract

**Background:**

Lung adenocarcinoma (LUAD) exhibits molecular heterogeneity, with mitochondrial damage affecting progression. The relationship between mitochondrial damage and immune infiltration, and Weighted Gene Co‐expression Network Analysis (WGCNA)‐derived biomarkers for LUAD classification and prognosis, remains unexplored.

**Aims:**

The objective of our research is to identify gene modules closely related to the clinical stages of LUAD using the WGCNA method. Based on the genes within these modules, we constructed machine learning (ML) models for classification and prognosis prediction, thereby facilitating precise diagnosis and personalized treatment of LUAD.

**Materials & Methods:**

Using GeneCards and The Cancer Genome Atlas (TCGA) databases, we screened differentially expressed mitochondrial damage‐related genes in LUAD. Immune cell infiltration patterns were assessed using Single‐Sample Gene Set Enrichment Analysis (SSGSEA) method. Functional enrichment analyses were conducted to explore biological functions and signaling pathways. Gene modules related to clinical stages of LUAD were identified by WGCNA. ML models were constructed for classification and prognosis prediction, and validated in an independent Gene Expression Omnibus (GEO) dataset.

**Results:**

The study revealed a significant relationship between mitochondrial damage and immune infiltration in LUAD. We identified a gene module closely associated with the clinical stages of LUAD. The ML models for classification and prognosis that were constructed demonstrated good effectiveness and generalization capabilities.

**Discussion:**

Mitochondrial damage‐related genes are crucial in LUAD progression and linked to immune infiltration. The gene module and models identified have potential applications in LUAD classification and prognosis, offering novel markers for precision medicine.

**Conclusion:**

This study uncovers the relationship between mitochondrial damage and immune infiltration in LUAD, paving the way for molecular classification, prognosis prediction, and personalized treatment strategies.

## 1 Introduction

Lung adenocarcinoma (LUAD), as a primary pathological subtype of lung cancer, encompasses intricate genetic and epigenetic alterations that underpin various cellular processes such as proliferation, apoptosis, and metabolism [1, 2]. These alterations not only modulate the fundamental biology of tumor cells but also profoundly reshape the composition of the tumor microenvironment, particularly the infiltration patterns of immune cells [3]. With the advancement of high‐throughput sequencing technologies in recent years, our understanding of the molecular mechanisms underlying LUAD has significantly deepened; however, achieving precise classification and individualized treatment strategies for LUAD at the molecular level remains a formidable challenge in clinical practice.

Mitochondria, the “powerhouses” of cells, play pivotal roles in maintaining cellular homeostasis and responding to external stresses [4]. Nevertheless, in tumors, mitochondria often exhibit damage, manifesting as mitochondrial DNA mutations, morphological alterations, metabolic reprogramming, and autophagic dysregulation [5, 6, 7, 8]. These changes not only affect the energy supply and metabolic pathways of tumor cells but also promote tumorigenesis and progression through the regulation of apoptosis and immune responses. Consequently, the expression patterns of mitochondrial damage–related genes in LUAD and their relationships with immune cell infiltration emerge as crucial entry points for investigating the molecular mechanisms of LUAD.

This study focuses on exploring the immune infiltration characteristics of mitochondrial damage‐related genes in LUAD and evaluating their potential as biomarkers for disease classification and prognosis. By integrating resources from the GeneCards and the Cancer Genome Atlas (TCGA) databases, we first screened out specifically differentially expressed mitochondrial damage‐related genes in LUAD. Subsequently, we employed single‐sample gene set enrichment analysis (SSGSEA) to assess the infiltration patterns of immune cells in LUAD, elucidating the relationship between immune cell infiltration and mitochondrial damage. Furthermore, we conducted Gene Ontology (GO) enrichment analysis, Kyoto Encyclopedia of Genes and Genomes (KEGG) enrichment analysis, and Gene Set Enrichment Analysis (GSEA) enrichment analyses to explore the biological functions and signaling pathways of these genes, deepening our understanding of the molecular mechanisms of LUAD.

To gain a more precise understanding of the roles of these genes in LUAD classification and prognosis assessment, we employed Weighted Gene Co‐expression Network Analysis (WGCNA) to meticulously identify a gene module among mitochondrial‐related genes that is tightly correlated with clinical stages of LUAD. Based on this pivotal module, we successfully constructed a machine learning classification model, which demonstrated robust effectiveness and generalization ability in the task of LUAD classification. Subsequently, we further validated the classification accuracy of the optimal model in an independent validation set from the Genomics Expression Omnibus (GEO) database. Moreover, we established a total survival prognosis model related to mitochondrial damage in LUAD and verified its validity in an external validation set from the GEO database. These efforts aim to contribute novel molecular markers, classification, and prognosis strategies to the field of precision medicine for LUAD.

In summary, this study has unveiled the intricate relationship between mitochondrial damage and immune infiltration in LUAD, while simultaneously opening up new avenues for the development of molecular classification, prognosis prediction, and individualized treatment strategies for LUAD.

## 2 Materials and Methods

### 2.1 Data Collection and Processing

#### 2.1.1 GeneCards Database

In the GeneCards database, the top 5000 most relevant genes were screened and obtained using keywords related to mitochondrial damage, serving as the input genes for WGCNA analysis.

#### 2.1.2 TCGA Database

The RNA‐Seq data, comprising 598 LUAD samples and 59 adjacent non‐tumor tissue samples, have been downloaded from the TCGA database. Among the clinical staging distribution, 298 cases are identified as Stage I, 123 as Stage II, 85 as Stage III, and 26 as Stage IV. Furthermore, 23 unique sets of data, derived from different time points or tissues of the same patient, are included in the dataset.

#### 2.1.3 GEO Database

GSE40419 from GEO was employed as an external validation set for the classification model. This dataset is well‐documented, containing RNA‐Seq data and clinical information derived from 87 LUAD tissues and 77 adjacent normal tissues. The GEO dataset GSE30219 was utilized as an external validation set for the prognostic model, offering RNA expression data and clinical outcome information from 293 lung tumor samples and 14 non‐tumor lung samples. This dataset provides a valuable independent resource for verifying the predictive accuracy and generalization capability of the model.

### 2.2 The Intersection Is Presented Using a Venn Diagram

To identify genes that are specifically expressed in LUAD and associated with mitochondrial damage, an intersection analysis was performed between the mitochondrial damage–related genes screened from GeneCards and the differentially expressed genes in TCGA‐LUAD. This step aimed to narrow down the research scope, focusing on genes that both significantly vary in LUAD and are intimately related to mitochondrial damage.

### 2.3 Immune Infiltration Analysis

#### 2.3.1 SSGSEA Method

In this study, the relationship between specific gene sets and immune cell infiltration in LUAD was investigated using the ssGSEA method from the GSVA package (version 1.44.5) within the R language environment.

#### 2.3.2 Feature Selection Using Random Forest Algorithm

To identify the immune cell types that contribute most significantly to the immune infiltration patterns in LUAD, this study employed the RF algorithm for feature selection on the SSGSEA results. Based on the ranking of feature importance, the top 10 crucial immune cell types were selected.

### 2.4 Differential Expression Analysis

In conducting differential expression analysis, the primary tool utilized was the DESeq2 software package (version 1.36.0) within the R programming language (version 4.2.1), with edgeR (version 3.38.2) serving as an auxiliary tool. Following the standard protocol of DESeq2, raw count data were processed with the aim of identifying genes exhibiting significant differences in expression under various conditions. Additionally, the variance stabilizing transformation (VST) method inherent in DESeq2 was applied to normalize the raw count matrix.

### 2.5 GO, KEGG Enrichment Analysis, and GSEA Analysis

To gain a comprehensive understanding of the biological functions and signaling pathways involved in the differentially expressed genes (DEGs) related to mitochondrial damage, we employed a suite of integrated bioinformatics analysis approaches. Within the R environment (version 4.2.1), we leveraged the clusterProfiler package (version 4.4.4) to conduct GO and KEGG enrichment analyses. The GO analysis unveiled the functional roles of genes across three dimensions: molecular function, cellular component, and biological process. Concurrently, the KEGG analysis illuminated the participation of these genes in metabolic pathways and signaling networks. Furthermore, we utilized the GOplot package (version 1.0.2) to visualize the GO enrichment results. Additionally, we performed GSEA, utilizing the clusterProfiler package in conjunction with the msigdbr package to access the c2.cp.all.v2022.1.Hs.symbols.gmt reference gene set from MSigDB Collections, which encompasses 3050 canonical pathways. This analysis evaluated the enrichment of DEGs within the predefined gene sets. Throughout the analysis process, we ensured the consistency and accuracy of all gene identifiers, performing necessary ID conversions with the org.Hs.eg.db package. This series of analyses collectively provided us with profound insights into the biological functions and signaling pathways of the DEGs associated with mitochondrial damage.

### 2.6 WGCNA Analysis

To identify the mitochondrial damage–related gene modules highly correlated with the clinical stages of LUAD, the WGCNA method was employed. WGCNA constructs a gene network, discerns highly correlated gene modules, and evaluates the associations between these modules and clinical information. Based on the correlation between modules and clinical stages, the key modules and their constituent genes were determined.

After rigorous preprocessing of the expression data, a range of soft‐thresholding powers, spanning from 1 to 10 and sequentially from 12 to 20 with a step size of 2, were selected to optimize the network topology. The pickSoftThreshold function was utilized to assess the goodness of fit (Signed *R* ^2^) and average connectivity of the scale‐free topology model across these powers. Relationship plots were generated depicting the fit of the scale‐free topology model versus the soft‐threshold values, as well as the relationship between average connectivity and soft‐threshold values, facilitating the selection of the optimal soft‐threshold, denoted as sft$powerEstimate.

After selecting the optimal soft‐threshold, a gene co‐expression network was constructed using the blockwiseModules function, considering an unsigned topological overlap matrix (TOM). A minimum module size of 30 genes was set. Genes were assigned to modules through the Dynamic Tree Cut algorithm, with the network structure optimized accordingly. The TOMs were preserved for subsequent analyses. Upon completion, the number of genes in each module was tallied, color labels were assigned to the modules, and dendrograms and color plots were generated to visually represent the organizational structure and similarity relationships of the gene modules.

To further delve into the characteristics of each module, module eigengenes (MEs) were extracted, and their expression matrices were accessed through net$MEs. The names of the MEs were then renamed using the stringr package to better align with their corresponding module color labels. Subsequently, the MEs were ordered using the orderMEs function to facilitate subsequent functional analyses. Pearson correlation coefficients and corresponding *p*‐values were calculated to evaluate the significance of the correlations between the MEs and phenotypic traits. Additionally, the module numbers to which specific genes belonged within the network were identified, and lists of all genes within those modules were retrieved, providing crucial gene sets for subsequent analyses.

### 2.7 Construction, Evaluation, and External Validation of a Mitochondrial Damage–Related LUAD Classification Model

#### 2.7.1 Construction and Evaluation of a Mitochondrial Damage–Related LUAD Classification Model

From the WGCNA results, genes within the GREY60 module were screened out as candidate key genes. These were further intersected with genes known to be associated with the prognosis of LUAD to identify crucial variables. Subsequently, six machine learning models for LUAD classification were constructed using these crucial variables. We selected a diverse range of algorithms including Naive Bayes, Decision Trees (implemented through rpart), k‐Nearest Neighbors (using kknn), RF (based on ranger), XGBoost, and Support Vector Machines (SVM). Each algorithm was configured to output probability predictions, and its performance was assessed through 10‐fold cross‐validation. During the evaluation process, we not only calculated the classification error rate (classif.ce) and the area under the curve (AUC, i.e., classif.auc), but also conducted a thorough understanding of the classification capabilities of the models through the visualization analysis of receiver operating characteristic (ROC) curves and precision‐recall (PRC) curves, along with their respective area under the curve (PR AUC).

To systematically compare the performance of different learners, we executed a benchmark test encompassing all selected learners under the same cross‐validation setup. By automating the benchmark test and aggregating the results, we focused on the AUC and PR AUC values obtained from both training and test sets, allowing for a comprehensive and insightful comparison of the performance of different learners on the classification task.

#### 2.7.2 External Validation of a Mitochondrial Damage–Related LUAD Classification Model

To validate the classification capability of the model, GSE40419 from the GEO database was utilized as an external validation set, containing 87 LUAD and 77 normal RNA‐Seq data points. Following the preprocessing and evaluation procedures adopted for the training set, the model underwent rigorous validation on this external dataset. By comparing the model's performance on the external validation set with that on the training and internal test sets, a comprehensive assessment of the model's stability and generalization ability was conducted.

### 2.8 Construction, Evaluation, and External Validation of a Mitochondrial Damage–Related LUAD Prognostic Model

#### 2.8.1 Construction and Evaluation of a Mitochondrial Damage–Related LUAD Prognostic Model

The mitochondrial damage–related LUAD dataset was partitioned through random sampling, with 60% designated as the training set and 40% as the test set. A classification model was constructed using the RF algorithm, where parameters were determined (specifically, 400 trees), and the top 30 key biomarkers were selected. Subsequently, an intersection of these biomarkers with a statistically significant (*p* < 0.01) set of molecules associated with prognosis was obtained. Based on this intersection, a Cox proportional hazards model (both univariate and multivariate) was employed to develop a total survival prognostic model for mitochondrial damage–related LUAD. For each sample, a risk score was calculated using the formula: Risk Score = Σ (Regression Coefficient) × (Expression Value of Key mRNA). Utilizing the median risk score as a threshold, patients were stratified into high‐ and low‐risk groups, and a K‐M survival analysis (with *p* < 0.01 considered significant) was conducted to validate the effectiveness of the model. Furthermore, the sensitivity and specificity of the model were evaluated using “timeROC,” and the differential expression of risk factors and key molecules was visualized with the ggplot2 package.

#### 2.8.2 External Validation of a Mitochondrial Damage–Related LUAD Prognostic Model

Using the same cutoff values and risk score calculation formula as the training set, the validity of the model on the GSE30219 validation set was verified through K‐M survival analysis (with *p* < 0.01 set as the significance level). Additionally, to comprehensively evaluate the model's performance, “timeROC” was employed to assess its sensitivity and specificity, and the ggplot2 package was utilized to visually present the differential expression of risk factors and key molecules.

## 3 Result

**FIGURE 1:**
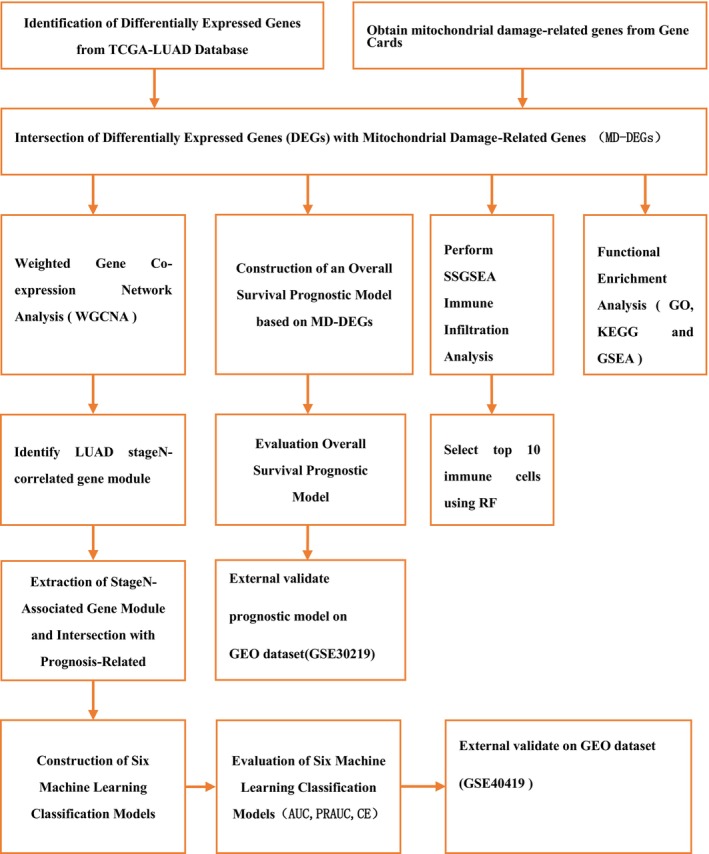
Flowchart of this study.

A flowchart for analyses is presented in Figure 1. The baseline characteristics are presented in Table 1.

**TABLE 1:** Detailed baseline characteristics of TCGA, GSE40419, and GSE30219 datasets.

Attribute	TCGA dataset (LUAD)	GEO GSE40419	GEO GSE30219
Dataset type	Cancer Genome Atlas (TCGA)	Gene Expression Omnibus (GEO)	Gene Expression Omnibus (GEO)
Disease focus	Lung adenocarcinoma (LUAD)	Lung adenocarcinoma (LUAD)	Lung adenocarcinoma (LUAD)
Sample type	RNA‐Seq	RNA‐Seq	Microarray
Number of tumor samples	598	87	293
Number of non‐tumor	59 (adjacent non‐tumor tissue)	77 (adjacent normal tissue)	14 (non‐tumor lung samples)
Clinical staging	Stage I: 298 Stage II: 123 Stage III: 85 Stage IV: 26	Not specified	Not specified

### 3.1 Differential Expression Analysis

**FIGURE 2:**
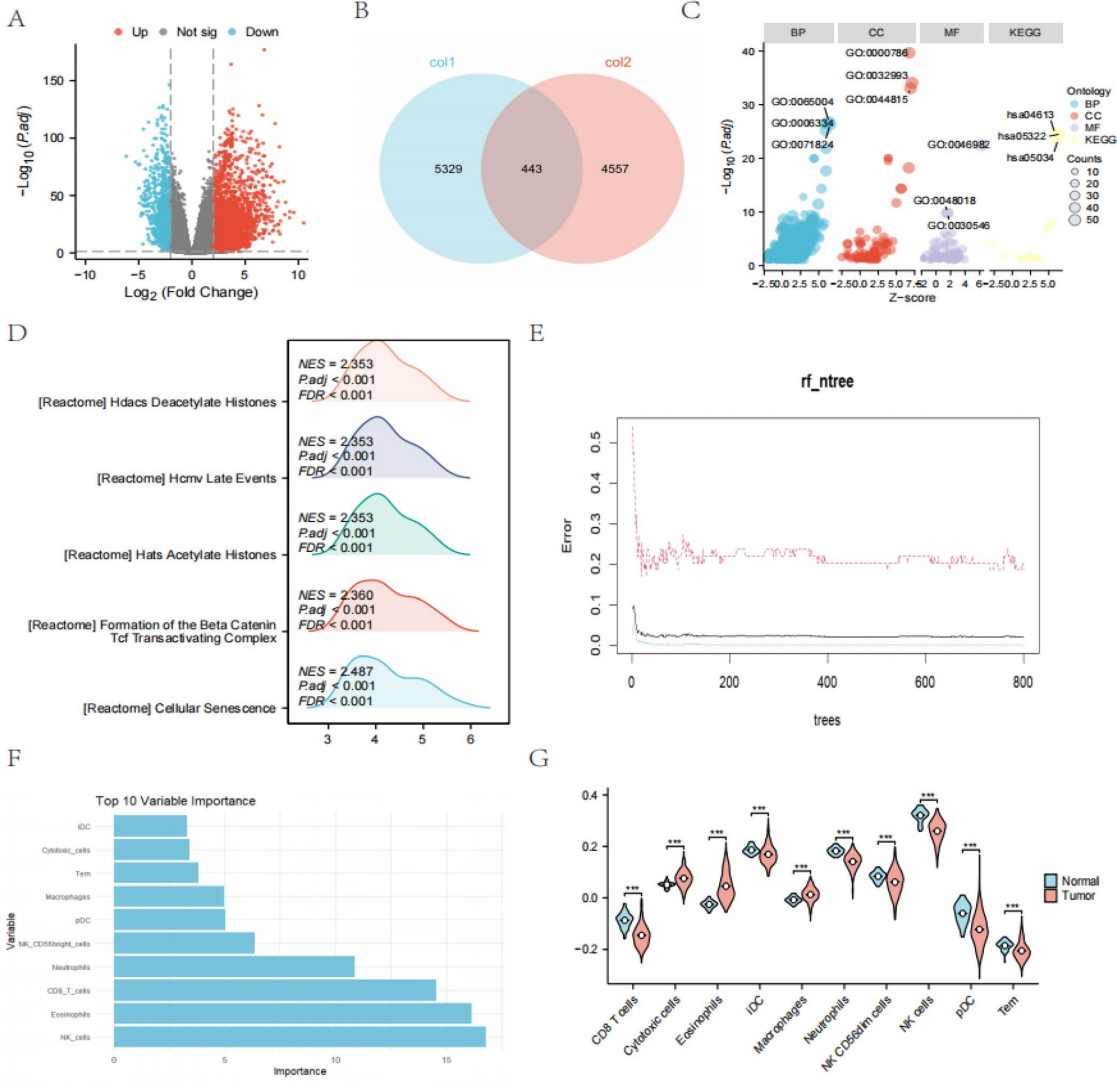
Differential expression analysis, immune infiltration analysis, and functional enrichment analysis. (A) volcano plot illustrating the DEGs, with red dots highlighting upregulated genes and blue dots representing downregulated genes. (B) The intersection between differentially expressed genes and mitochondrial damage‐associated genes. (C) GO and KEGG analysis. (D) GSEA analysis. (E) The relationship between the classification error rate and the number of trees in the Random Forests algorithm. (F) The importance of these 10 key immune cell types, with the horizontal axis representing different immune cell types and the vertical axis indicating their respective importance scores. (G) The differential expression of these 10 immune cells between LUAD and adjacent normal tissues in the form of a violin plot. The blue section represents the expression levels in normal tissues, while the red section denotes those in LUAD tissues. The symbol *** represents P < 0.001, ** represents P < 0.01, and * represents P < 0.05.

This study identified 5803 significantly differentially expressed coding genes through the application of the screening criteria of |logFC| > 2 and *p*.adj < 0.01. Specifically, among these 5803 genes, 4859 exhibited significant upregulation (logFC > 2 and *p*.adj < 0.01), while the remaining 944 genes demonstrated significant downregulation (logFC< −2 and *p*.adj < 0.01). Figure 2A presents a volcano plot illustrating these DEGs, with red dots highlighting upregulated genes and blue dots representing downregulated genes.

### 3.2 GO, KEGG, and GSEA Analysis

Figure 2B depicts the intersection between differentially expressed genes and mitochondrial damage–associated genes, revealing a total of 443 differentially expressed genes related to mitochondrial damage in LUAD. We performed functional enrichment analysis on these genes. The analysis results indicate significant enrichment of multiple crucial functions and pathways under the significance threshold of *p*.adj < 0.1. In biological processes (BP), DNA assembly, nucleosome formation, and chromatin assembly play pivotal roles in gene expression regulation and cell cycle progression. At the cellular component (CC) level, nucleosomes and DNA packaging complexes maintain genomic stability and cellular architecture. In terms of molecular functions (MF), protein heterodimerization and receptor activity are essential for intercellular communication, growth regulation, and metabolic control. Furthermore, KEGG pathway analysis reveals the potential involvement of these molecules in specific diseases, such as neutrophil extracellular trap (NET) formation, systemic lupus erythematosus (SLE), alcoholism, and viral carcinogenesis, as well as in biological processes; as illustrated in Figure 2C and detailed in Table 2.

**TABLE 2:** GO, KEGG, and GSEA analysis.

Ontology	ID	Description	Gene ratio	Bg ratio	*p*	*p*.adjust	*z* score
BP	GO:0065004	Protein‐DNA complex assembly	45/438	203/18800	3.77e‐31	1.71e‐27	6.4101
BP	GO:0006334	Nucleosome assembly	37/438	126/18800	1.35e‐30	3.06e‐27	5.754
BP	GO:0071824	Protein‐DNA complex subunit organization	47/438	237/18800	3.5e‐30	5.28e‐27	6.5639
BP	GO:0034728	Nucleosome organization	39/438	159/18800	6.68e‐29	7.57e‐26	5.9247
BP	GO:0031497	Chromatin assembly	40/438	205/18800	1.58e‐25	1.43e‐22	6.0083
CC	GO:0000786	Nucleosome	46/438	129/19594	4.72e‐43	2.08e‐40	6.7823
CC	GO:0032993	Protein‐DNA complex	51/438	220/19594	4.19e‐37	9.22e‐35	7.1414
CC	GO:0044815	DNA packaging complex	48/438	198/19594	6.15e‐36	9.02e‐34	6.9282
CC	GO:0043505	CENP‐A containing nucleosome	15/438	18/19594	1.05e‐22	9.26e‐21	3.873
CC	GO:0061638	CENP‐A containing chromatin	15/438	18/19594	1.05e‐22	9.26e‐21	3.873
MF	GO:0046982	Protein heterodimerization activity	50/433	332/18410	4.58e‐26	3.33e‐23	6.2225
MF	GO:0048018	Receptor ligand activity	42/433	489/18410	4.25e‐13	1.54e‐10	1.543
MF	GO:0030546	Signaling receptor activator activity	42/433	496/18410	6.82e‐13	1.65e‐10	1.543
MF	GO:0005179	Hormone activity	17/433	122/18410	4.03e‐09	7.33e‐07	2.1828
MF	GO:0008083	Growth factor activity	19/433	162/18410	9.21e‐09	1.34e‐06	1.1471
KEGG	hsa04613	Neutrophil extracellular trap formation	49/314	190/8164	8.47e‐28	2.35e‐25	5.8571
KEGG	hsa05322	Systemic lupus erythematosus	42/314	136/8164	2.43e‐27	3.36e‐25	6.4807
KEGG	hsa05034	Alcoholism	47/314	187/8164	3.87e‐26	3.58e‐24	6.2722
KEGG	hsa05203	Viral carcinogenesis	30/314	204/8164	1.69e‐10	1.17e‐08	5.4772
KEGG	hsa04110	Cell cycle	22/314	126/8164	1.94e‐09	1.07e‐07	4.6904

Under statistical significance criteria (adjusted *p*‐value < 0.05 and false discovery rate (FDR) or *q*‐value < 0.25), we identified 82 significantly enriched gene sets. Specifically, we uncovered gene sets related to cellular senescence (reactome_cellular_senescence), formation of the β‐catenin/tcf transcription factor complex (reactome_formation_of_the_beta_catenin_tcf_tran…), histone acetylation (reactome_hats_acetylate_histones), late events in the human cytomegalovirus (hcmv), life cycle (reactome_hcmv_late_events), histone deacetylation (reactome_hdacs_deacetylate_histones), rho gtpase activation of protein kinases (reactome_rho_gtpases_activate_pkns), post‐translational protein modification (reactome_post_translational_protein_modification), and systemic lupus erythematosus (kegg_systemic_lupus_erythematosus); as illustrated in Figure 2D and detailed in Table 3.

**TABLE 3:** GSEA analysis.

ID	Set size	Enrichment score	NES	*p*	*p*.adjust	*q* value
REACTOME_CELLULAR_SENESCENCE	49	0.4702962	2.487284	4.85e‐06	0.0006	0.0003
REACTOME_FORMATION_OF_THE_BETA_CATENIN_TCF_TRANSACTIVATING_COMPLEX	38	0.4825159	2.360270	2.94e‐05	0.0006	0.0003
REACTOME_HATS_ACETYLATE_HISTONES	42	0.4677223	2.353044	4.4e‐05	0.0006	0.0003
REACTOME_HCMV_LATE_EVENTS	42	0.4677223	2.353044	4.4e‐05	0.0006	0.0003
REACTOME_HDACS_DEACETYLATE_HISTONES	42	0.4677223	2.353044	4.4e‐05	0.0006	0.0003
REACTOME_RHO_GTPASES_ACTIVATE_PKNS	38	0.4794787	2.345413	3.86e‐05	0.0006	0.0003
REACTOME_POST_TRANSLATIONAL_PROTEIN_MODIFICATION	74	0.4076633	2.320825	2.75e‐05	0.0006	0.0003
KEGG_SYSTEMIC_LUPUS_ERYTHEMATOSUS	43	0.4534848	2.306063	7.03e‐05	0.0006	0.0003
REACTOME_HCMV_EARLY_EVENTS	46	0.4434346	2.301366	7.23e‐05	0.0006	0.0003
REACTOME_HCMV_INFECTION	46	0.4434346	2.301366	7.23e‐05	0.0006	0.0003

### 3.3 Key Immune Cell Types and Their Differential Expression in LUAD Versus Normal Tissues

We performed feature selection on SSGSEA data using the RF algorithm. These immune cell types include CD8 T cells, cytotoxic cells, eosinophils, immature dendritic cells (iDCs), macrophages, neutrophils, CD56dim natural killer cells (NK CD56dim cells), natural killer cells (NK cells), plasmacytoid dendritic cells (pDCs), and effector memory T cells (Tem). Figure 2E illustrates the relationship between the classification error rate and the number of trees in the RF algorithm. Figure 2F showcases the importance of these 10 key immune cell types, with the horizontal axis representing different immune cell types and the vertical axis indicating their respective importance scores. Figure 2G presents the differential expression of these 10 immune cells between LUAD and adjacent normal tissues in the form of a violin plot. The blue section represents the expression levels in normal tissues, while the red section denotes those in LUAD tissues. Notably, there exists a statistically significant difference (*p* < 0.01) in the expression of these 10 immune cells between the two groups.

### 3.4 WGCNA Analysis

**FIGURE 3:**
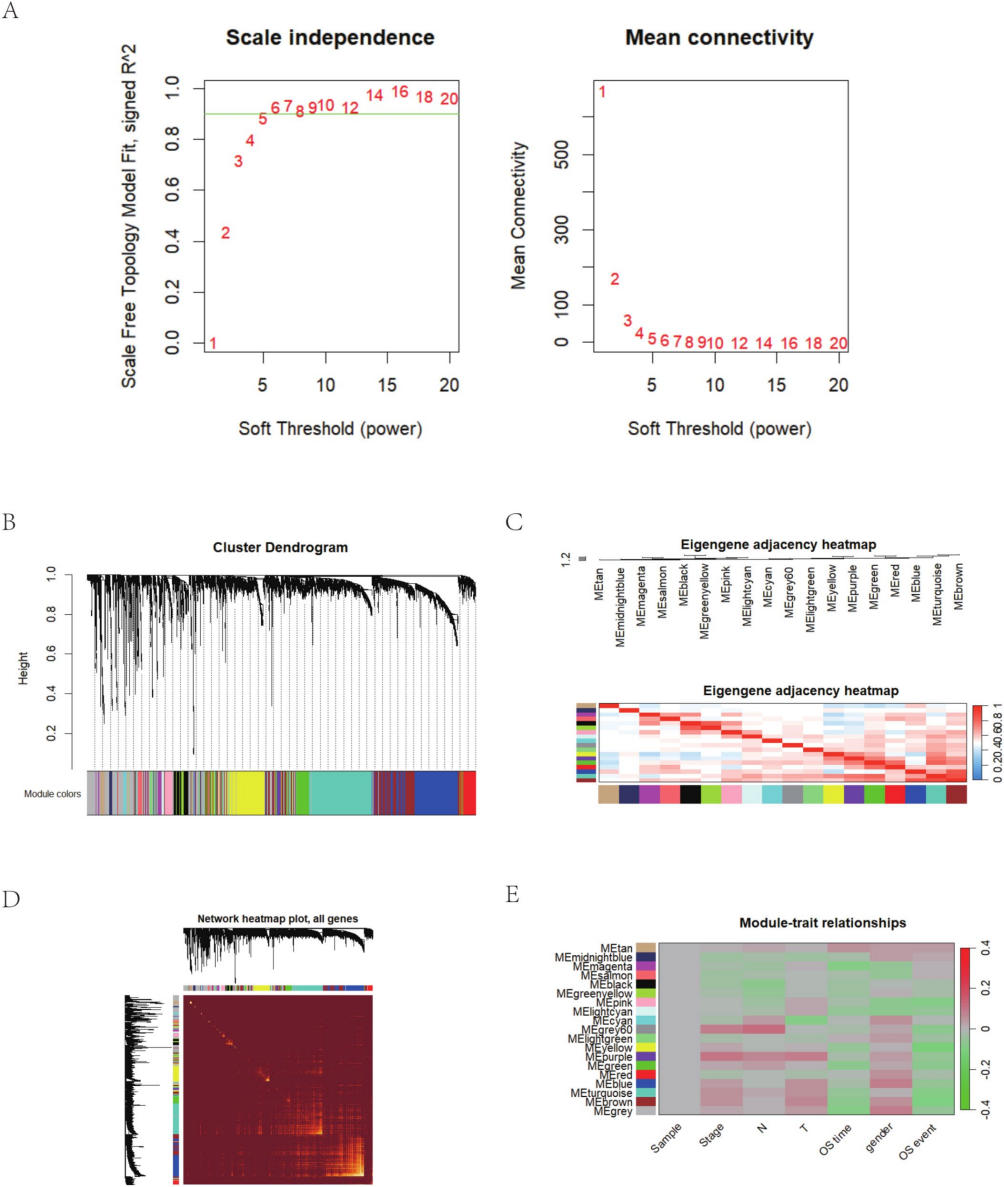
WGCNA analysis. (A) Signed *R* ^2^ variation with power, aiming for 0.90. (B) The hierarchy and color distribution of the modules. (C) The correlations between modules. (D) A visual representation of these modules' structures and interconnectivities. (E) The correlations between modules and external features.

Analysis showed that Power 5 provided the best soft‐thresholding with an SFT.R.sq. of 0.887 and a slope near −1.71, indicating good network properties. Figure 3A demonstrates the variation of signed *R* ^2^ along with the change in power, as well as the relationship between average connectivity and power. Using the optimal soft‐thresholding power, we constructed a stable gene co‐expression network and streamlined the modules and color codes. Figure 3B displays the hierarchy and color distribution of the modules. Figure 3C reveals the correlations between modules. Figure 3D presents the TOM plot, which is utilized to exhibit the intricate relationships among genes. The shading intensity in the plot indicates the degree of correlation or topological overlap between genes. Additionally, Figure 3E has delved into the correlations between modules and external features.

### 3.5 Construction and Evaluation of a Machine Learning Classification Model for LUAD Associated With Mitochondrial Damage

#### 3.5.1 Construction and Evaluation of a Machine Learning Classification Model

**FIGURE 4:**
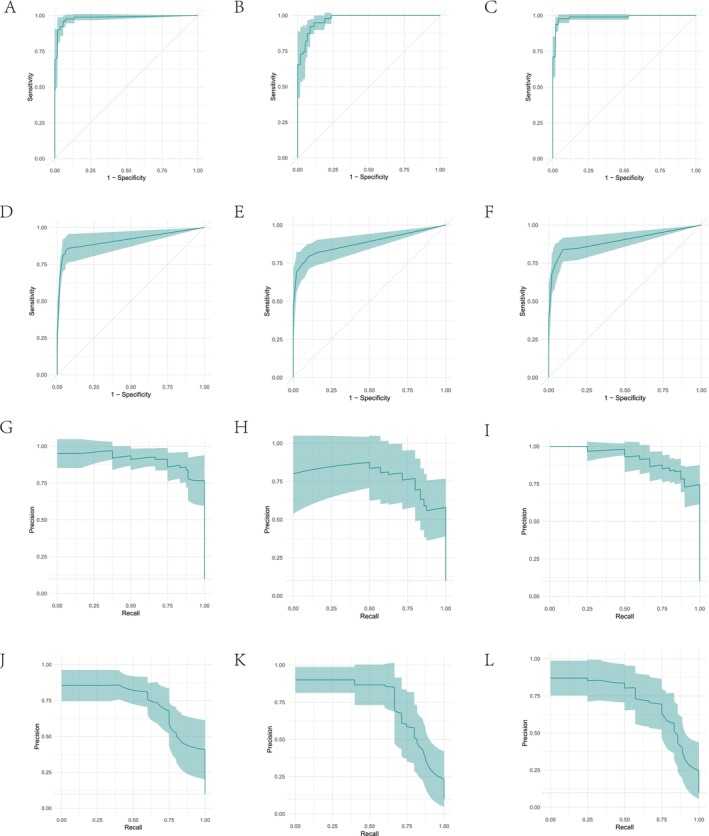
Evaluating AUC and PRAUC of six machine learning algorithms via 10‐fold cross‐validation. (A–F) AUC of six machine learning algorithms via 10‐fold cross‐validation. (G–L) PRAUC of six machine learning algorithms via 10‐fold cross‐validation.

We employed the 10‐fold cross‐validation (CV) method to evaluate the performance of six classification algorithms on the dataset. The evaluation metrics included training set accuracy (AUC_Train) and test set accuracy (AUC_Test). The results indicated that the classif.ranger algorithm achieved an accuracy of 1.0000000 on the training set and 0.9863251 on the test set, demonstrating its robust performance and generalization ability. The classif.svm and classif.naive_bayes algorithms achieved test set accuracies of 0.9776699 and 0.9732473, respectively. Meanwhile, the classif.kknn algorithm had a test set accuracy of 0.9617538. The classif.rpart and classif.xgboost algorithms exhibited test set accuracies of 0.8514950 and 0.8697466, respectively. Overall, the classif.ranger algorithm performed the most outstandingly in this study. The detailed results are shown in Table 4. The AUC curves for the six algorithms are presented in Figure 4A–F.

**TABLE 4:** Performance comparison of classification algorithms based on AUC values on the training and test sets.

Learner ID	Resampling ID	Iters	AUC_Train	AUC_Test
classif.kknn	cv	10	0.9989598	0.9617538
classif.naive_bayes	cv	10	0.9814279	0.9732473
**classif.ranger**	**cv**	**10**	**1.0000000**	**0.9863251**
classif.rpart	cv	10	0.9411919	0.8514950
classif.svm	cv	10	0.9856478	0.9776699
classif.xgboost	cv	10	0.9328295	0.8697466

*Note:* The optimal model is displayed in bold font.

We employed the 10‐fold cross‐validation (CV) method to evaluate the performance of six classification algorithms on the dataset. The evaluation metrics included training set precision‐recall area under curve (PRAUC_Train) and test set precision‐recall area under curve (PRAUC_Test). The results indicated that the classif.ranger algorithm achieved a PRAUC of 1.0000000 on the training set and 0.9003509 on the test set, demonstrating its robust performance and generalization ability. The classif.svm and classif.naive_bayes algorithms achieved test set PRAUCs of 0.8349373 and 0.8085212, respectively. Meanwhile, the classif.kknn algorithm had a test set PRAUC of 0.8794921. The classif.rpart and classif.xgboost algorithms exhibited test set PRAUCs of 0.6096765 and 0.6533493, respectively. Overall, the classif.ranger algorithm performed the most outstandingly in this study. The detailed results are shown in Table 5. The PRAUC curves for the six algorithms are presented in Figure 4G–L.

**TABLE 5:** PRAUC of classification algorithms on training and testing sets.

LEARNER_ID	RESAMPLING_ID	Iters	PRAUC_train	PRAUC_test
classif.kknn	cv	10	0.9912749	0.8794921
classif.naive_bayes	cv	10	0.8918983	0.8085212
**classif.ranger**	**cv**	**10**	**1.0000000**	**0.9003509**
classif.rpart	cv	10	0.8225831	0.6096765
classif.svm	cv	10	0.8709590	0.8349373
classif.xgboost	cv	10	0.8842174	0.6533493

*Note:* The optimal model is displayed in bold font.

**FIGURE 5:**
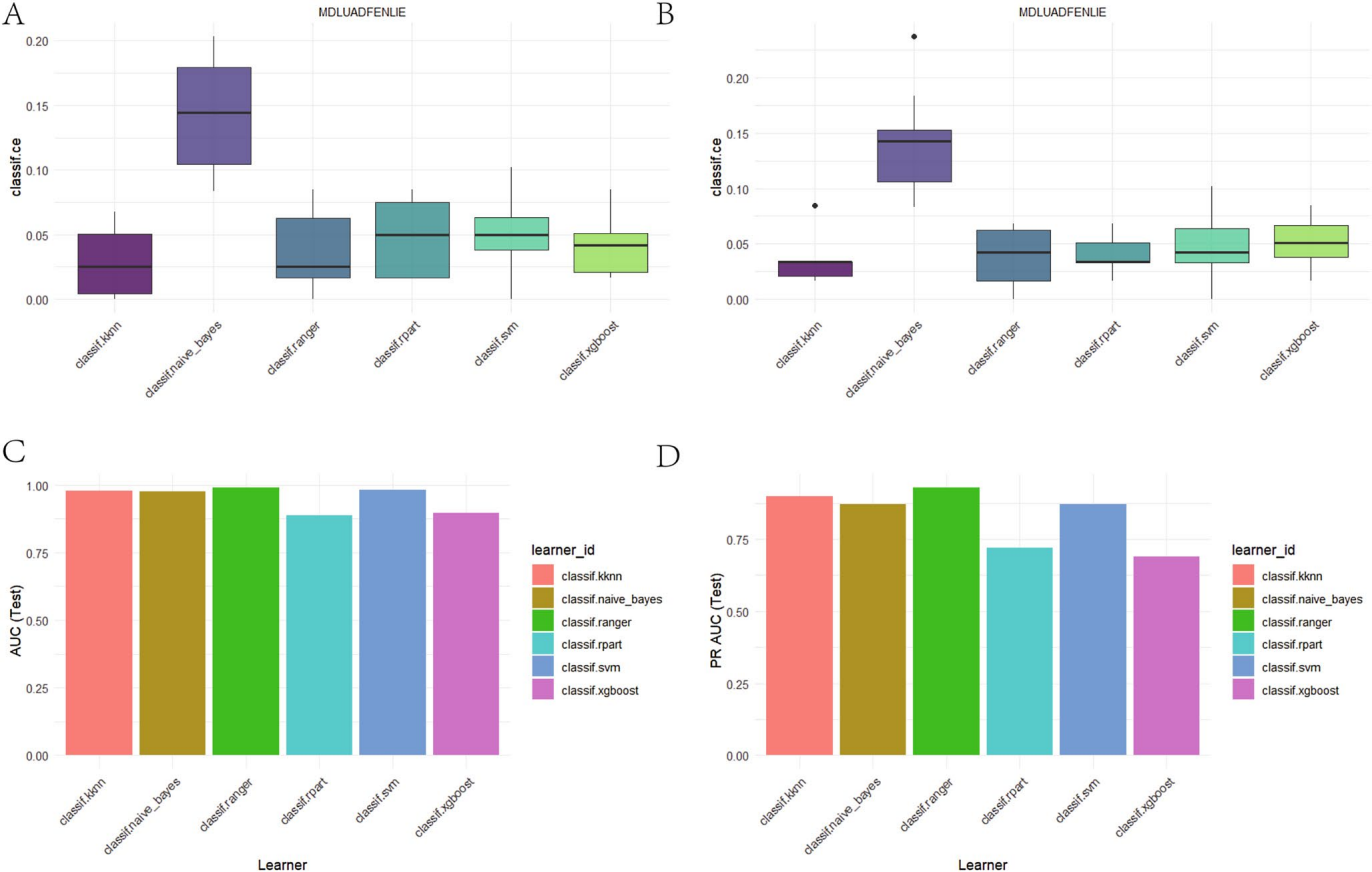
Comprehensive evaluation of model classification performance across AUC, PRAUC, and error rate. (A) Model performance: AUC error rate below 15%. (B) Consistent high AUC (> 80%) for robust classification. (C) PRAUC excels with error rate below 15%. (D) PRAUC area exceeds 72%, demonstrating robustness.

As shown in Figure 5A, when evaluated using AUC, the model's error rate is below 15%, indicating superior classification performance. Figure 5B demonstrates that when assessed with PRAUC, the model equally excels, with an error rate under 15%, proving its robust classification capabilities across different evaluation metrics.

As depicted in Figure 5C, when evaluated using AUC, the area under the AUC curve consistently exceeds 80%, signifying exceptional classification performance. Furthermore, Figure 5D reveals that the area under the PRAUC curve surpasses 72%, reinforcing the model's robust classification capabilities across diverse evaluation metrics.

#### 3.5.2 The Validation of Machine Learning Classification Models on an External Validation Set

**FIGURE 6:**
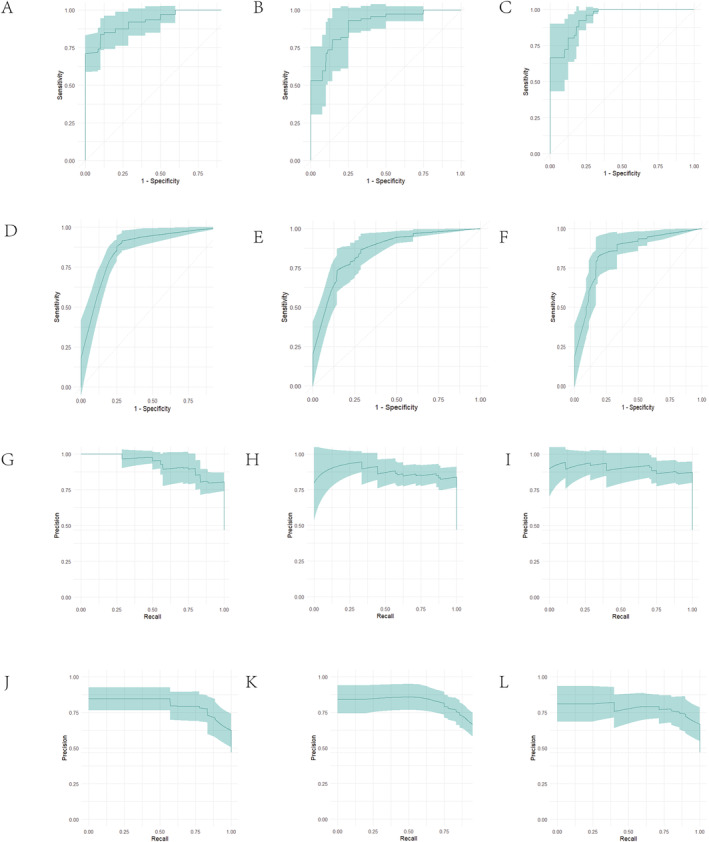
Evaluating AUC and PRAUC of six machine learning algorithms via 10‐fold cross‐validation on external validation sets. (A–F) AUC of six machine learning algorithms via 10‐fold cross‐validation on external validation sets. (G–L) PRAUC of six machine learning algorithms via 10‐fold cross‐validation on external validation sets.

During the evaluation of the machine learning model performance on an independent external validation set, we employed the same six classification algorithms as those used in the training set and conducted 10 iterations of cross‐validation tests. Table 6 presents the AUC performance metrics for each model on both the training set (AUC_Train) and the test set (AUC_Test). Specifically, the kknn model achieved an exceptionally high AUC value of 0.9987069 on the training set and maintained good performance with an AUC of 0.9306498 on the test set. The naive Bayes model demonstrated stable classification capability with similar performance on both the training (0.9227982) and test sets (0.9118006). The ranger model performed best on the test set, achieving an AUC of 0.9436310, slightly outperforming other models. In contrast, the rpart model showed a relatively lower AUC of 0.8762202 on the test set, though its performance on the training set was still good (0.9220902). The SVM and Xgboost models achieved AUCs of 0.9477133 and 0.8719444, respectively, on the test set, demonstrating their effectiveness and adaptability in classification tasks. Overall, these results demonstrate the effectiveness of the six machine learning models, particularly the ranger model, in handling external datasets. See Figure 6A–F for details.

**TABLE 6:** AUC of machine learning models on the external validation dataset.

Learner ID	Resampling ID	Iterations	AUC_Train	AUC_Test
classif.kknn	cv	10	0.9987069	0.9306498
classif.naive_bayes	cv	10	0.9227982	0.9118006
**classif.ranger**	**cv**	**10**	**0.9985986**	**0.9436310**
classif.rpart	cv	10	0.9220902	0.8762202
classif.svm	cv	10	0.9664842	0.9477133
classif.xgboost	cv	10	0.9707943	0.8719444

*Note:* The optimal model is displayed in bold font.

As shown in Table 7, we evaluated the classification performance of six machine learning models, with a focus on the PRAUC. The experimental results indicated that the ranger model excelled on the test set, achieving the highest PRAUC value of 0.9630286, demonstrating its robust generalization ability. The Kknn, Naive_bayes, and SVM models also achieved commendable PRAUC values on the test set, with 0.9499406, 0.9411524, and 0.9606777 respectively, validating their classification effectiveness. In contrast, the Rpart and Xgboost models exhibited slightly lower PRAUC values on the test set, but still retained certain application values. See Figure 6G–L for details.

**TABLE 7:** PRAUC of machine learning models on the external validation dataset.

Learner ID	Resampling ID	Iterations	PRAUC_Train	PRAUC_Test
classif.kknn	cv	10	0.9988466	0.9499406
classif.naive_bayes	cv	10	0.9456092	0.9411524
**classif.ranger**	**cv**	**10**	**0.9988001**	**0.9630286**
classif.rpart	cv	10	0.9288316	0.8965830
classif.svm	cv	10	0.9765841	0.9606777
classif.xgboost	cv	10	0.9753441	0.8897655

*Note:* The optimal model is displayed in bold font.

**FIGURE 7:**
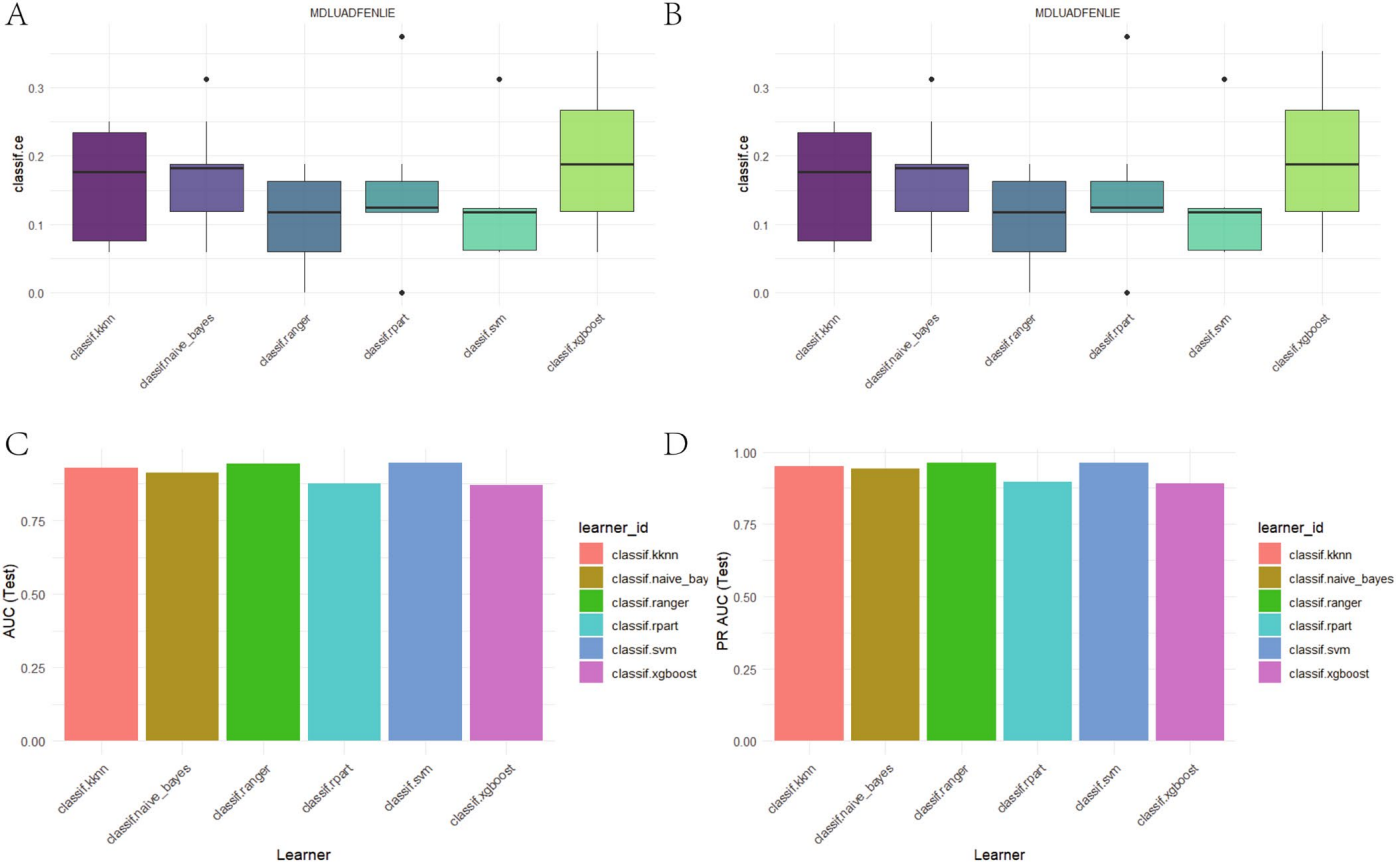
Comprehensive evaluation of model classification performance across AUC, PRAUC, and error rate on external validation sets. (A) Model performance: AUC error rate below 27%. (B) Consistent high AUC (> 88%) for robust classification. (C) PRAUC excels with error rate below 27%. (D) PRAUC area exceeds 88%, demonstrating robustness.

As shown in Figure 7A, when evaluated using AUC, the model's error rate is below 27%, indicating superior classification performance. Figure 7B demonstrates that when assessed with PRAUC, the model equally excels, with an error rate under 27%, proving its robust classification capabilities across different evaluation metrics.

As depicted in Figure 7C, when evaluated using AUC, the area under the AUC curve consistently exceeds 86%, signifying exceptional classification performance. Furthermore, Figure 7D reveals that the area under the PRAUC curve surpasses 88%, reinforcing the model's robust classification capabilities across diverse evaluation metrics.

### 3.6 Construction and Evaluation of Prognostic Model for LUAD Associated With Mitochondrial Damage

#### 3.6.1 Construction and Evaluation of a Prognostic Model

**FIGURE 8:**
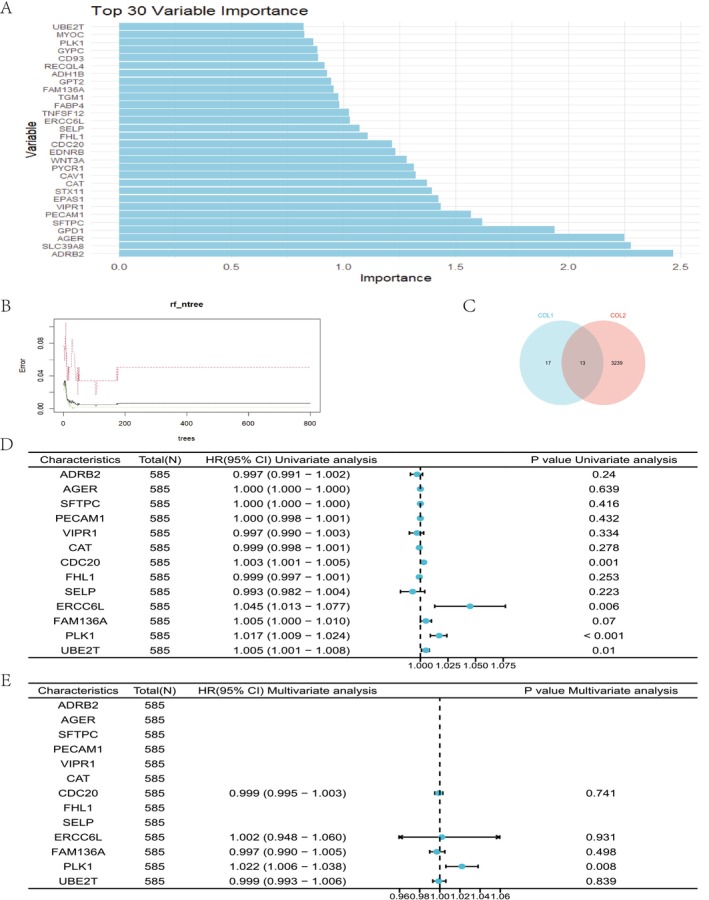
Construction prognostic model of mitochondrial damage–related in LUAD. (A) The ranked importance of the top 30 variables in the RF model, with the vertical axis representing the importance score and the horizontal axis indicating the variable names. (B) The relationship between the number of trees (or model complexity) and the model's error rate in the RF. The horizontal axis denotes the increasing number of trees, while the vertical axis reflects the corresponding error rate. (C) A venn diagram comparing the variables identified by the RF algorithm with those found to be significantly associated with the overall survival prognosis of LUAD. (D) Forest plot for univariate analyses. (E) Forest plot for multivariate analyses.

Through preliminary optimization using the RF algorithm, we developed a model comprising 400 trees, which achieved an excellent balance between performance and computational efficiency. This model exhibited remarkable performance on the training set, with an out‐of‐bag (OOB) error rate as low as 0.67%. Specifically, regarding classification outcomes, the misclassification rates for normal and tumor tissue samples were as low as 0.0508% and 0.001869%, respectively. Similarly, on the test set, the model maintained high accuracy, correctly classifying all samples without error. Ultimately, we identified 30 crucial molecules associated with mitochondrial damage‐related LUAD. Subsequently, applying a significance threshold of *p* < 0.01, we screened out 3252 mRNAs that were intimately correlated with the overall survival prognosis of LUAD. An intersection between these two sets yielded 13 common mRNAs. These 13 mRNAs were selected as independent variables for the COX prognostic model, which was constructed to evaluate the overall survival prognosis associated with mitochondrial damage‐related LUAD. Figure 8A presents the ranked importance of the top 30 variables in the RF model, with the vertical axis representing the importance score and the horizontal axis indicating the variable names. Figure 8B investigates the relationship between the number of trees (or model complexity) and the model's error rate in the RF. The horizontal axis denotes the increasing number of trees, while the vertical axis reflects the corresponding error rate. This analysis aids in identifying the optimal number of trees that balances both a low error rate and computational efficiency. Figure 8C displays a venn diagram comparing the variables identified by the RF algorithm with those found to be significantly associated with the overall survival prognosis of LUAD. Through this comparison, a final set of 13 common variables is obtained. Based on multivariate COX analysis, we have constructed a prognostic model for LUAD related to mitochondrial damage. Univariate and multivariate analyses were conducted on key genes related to mitochondrial damage to assess their association with overall survival prognosis in LUAD. A total of 585 cases were included in the analysis. In the univariate analysis, we observed that genes such as CDC20 (HR: 1.003, 95% CI: 1.001–1.005, *p* = 0.001), ERCC6L (HR: 1.045, 95% CI: 1.013–1.077, *p* = 0.006), FAM136A (HR: 1.005, 95% CI: 1.000–1.010, *p* = 0.070, approaching significance), PLK1 (HR: 1.017, 95% CI: 1.009–1.024, *p* < 0.001), and UBE2T (HR: 1.005, 95% CI: 1.001–1.008, *p* = 0.010) were associated with the overall survival prognosis of LUAD. However, in the multivariate analysis, only the PLK1 gene maintained its significance, with the HR increasing to 1.022 (95% CI: 1.006–1.038, *p* = 0.008), indicating that PLK1 is an independent prognostic factor in the studied model. See Table 8 for details. The bolded font represents a P < 0.05.

**TABLE 8:** Univariate and multivariate analysis.

Characteristics	Total (*N*)	Univariate analysis		Multivariate analysis	
		Hazard ratio (95% CI)	*p*	Hazard ratio (95% CI)	*p*
ADRB2	585	0.997 (0.991–1.002)	0.240		
AGER	585	1.000 (1.000–1.000)	0.639		
SFTPC	585	1.000 (1.000–1.000)	0.416		
PECAM1	585	1.000 (0.998–1.001)	0.432		
VIPR1	585	0.997 (0.990–1.003)	0.334		
CAT	585	0.999 (0.998–1.001)	0.278		
CDC20	585	1.003 (1.001–1.005)	**0.001**	0.999 (0.995–1.003)	0.741
FHL1	585	0.999 (0.997–1.001)	0.253		
SELP	585	0.993 (0.982–1.004)	0.223		
ERCC6L	585	1.045 (1.013–1.077)	**0.006**	1.002 (0.948–1.060)	0.931
FAM136A	585	1.005 (1.000–1.010)	0.070	0.997 (0.990–1.005)	0.498
PLK1	585	1.017 (1.009–1.024)	**< 0.001**	1.022 (1.006–1.038)	**0.008**
UBE2T	585	1.005 (1.001–1.008)	**0.010**	0.999 (0.993–1.006)	0.839

*Note:* The optimal model is displayed in bold font.

The model demonstrates good predictive power with a C‐index of 0.613 (95% CI: 0.590–0.636). The likelihood ratio, Wald, and score (logrank) tests were all significant (*p* < 0.01), validating the effectiveness of the model. The proportional hazards (PH) assumption test revealed that all variables had *p*‐values greater than 0.05, with an overall *p*‐value of 0.6286, indicating that the model satisfies the PH assumption, whereby the HRs of the variables remain constant over time, making Cox regression an appropriate analytical tool. Furthermore, the variance inflation factor (VIF) assessment confirmed the absence of severe multicollinearity among the variables (VIF < 10). Figure 8D,E respectively present the results of the univariate and multivariate analyses. This research contributes to the scientific understanding and clinical decision‐making process for LUAD, particularly in the context of mitochondrial damage. The prognostic model is constructed based on a linear combination of the expression levels of five mRNAs, with the weights derived from multivariate Cox regression coefficients. The formula for calculating the risk score is as follows: Risk Score = −0.1333–0.000672 × CDC20 + 0.002458 × ERCC6L–0.002570 × FAM136A + 0.021766 × PLK1–0.000650 × UBE2T. This formula was applied to compute the risk scores for patients in the training set. Subsequently, the median risk score served as the cutoff point to stratify patients into two distinct groups: a low‐risk group (*n* = 261) and a high‐risk group (*n* = 261).

**FIGURE 9:**
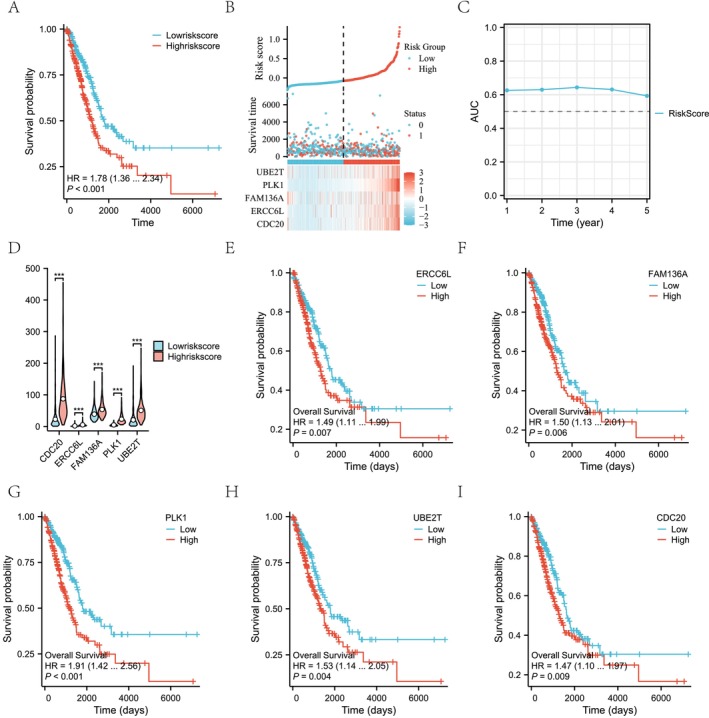
Evaluation prognostic model of mitochondrial damage‐related in LUAD. (A) K‐M survival analysis between low‐risk and high‐risk groups. (B) Risk score distribution and survival status between low‐risk and high‐risk groups. (C) Time‐dependent ROC analysis of the risk score model. (D) Differential expression analysis of five key genes of mitochondrial damage‐related between low‐risk and high‐risk groups. (E) K‐M survival analysis for the key molecule ERCC6L. (F) K‐M survival analysis for the key molecule FAM136A. (G) K‐M survival analysis for the key molecule PLK1. (H) K‐M survival analysis for the key molecule UBE2T. (I) K‐M survival analysis for the key molecule CDC20. The symbol *** represents P < 0.001, ** represents P < 0.01, and * represents P < 0.05.

The K‐M analysis (Figure 9A) revealed that the overall survival in the low‐risk group was superior to that in the high‐risk group (median OS: 1790 days vs. 1209 days), with a *p* value of < 0.001, validating the high accuracy of the model. Figure 9B illustrates the distribution of risk scores, survival status, and mRNA expression. An increase in risk scores was associated with heightened mortality and decreased survival rates among patients. Additionally, the heatmap demonstrates that the expression of the five molecules also intensified as the risk scores rose. As depicted in Figure 9C, utilizing the timeROC package, the RiskScore model exhibited a gradual decrease in optimal thresholds over the 1‐ to 5‐year follow‐up period, while maintaining stable sensitivity and specificity. Notably, the negative predictive value reached as high as 0.92379, highlighting the model's advantage in identifying low‐risk patients. Additionally, the AUC values fluctuated between 0.6 and 0.65, indicating that the RiskScore model possessed consistent predictive performance across various time points. Expression analysis (Figure 9D) revealed significantly elevated levels of CDC20, ERCC6L, FAM136A, PLK1, and UBE2T in the high‐risk group compared to the low‐risk group, suggesting a positive correlation between their overexpression and increased risk, potentially promoting LUAD progression.

We further conducted a survival analysis on the expression of five genes in TCGA‐LUAD patients. Based on the median mRNA expression of each gene signature, patients were stratified into high and low mRNA expression groups. The results demonstrated that high expression of ERCC6L (*p* = 0.007, HR = 1.49, Figure 9E), FAM136A (*p* = 0.006, HR = 1.50, Figure 9F), PLK1 (*p* < 0.001, HR = 1.91, Figure 9G), UBE2T (*p* = 0.004, HR = 1.53, Figure 9H), and CDC20 (*p* = 0.009, HR = 1.47, Figure 9I) was significantly associated with poorer prognosis. This finding is consistent with the heatmap presented in Figure 9B, indicating that in LUAD, high expression of CDC20, ERCC6L, FAM136A, PLK1, and UBE2T is correlated with adverse outcomes.

#### 3.6.2 Validation Prognostic Model of Mitochondrial Damage–Related LUAD on External Validation Sets

**FIGURE 10:**
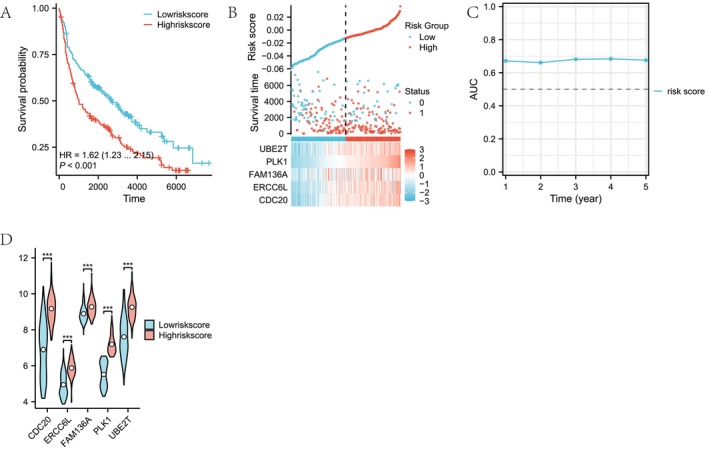
Validation prognostic model of mitochondrial damage–related in LUAD on external validation sets. (A) K‐M survival analysis between low‐risk and high‐risk groups. (B) Risk score distribution and survival status between low‐risk and high‐risk groups. (C) Time‐dependent ROC analysis of the prognostic model. (D) Differential expression analysis of five key genes of mitochondrial damage–related between low‐risk and high‐risk groups. The symbol *** represents P < 0.001, ** represents P < 0.01, and * represents P < 0.05.

In the validation set GSE31210, we applied the same coefficients as used in the training set for analysis. K‐M analysis (Figure 10A) revealed that patients in the low‐risk group exhibited superior overall survival compared to those in the high‐risk group (median OS: 2730 days for low‐risk vs. 1020 days for high‐risk), with a statistically significant difference (*p* < 0.001), validating the high accuracy of the model.

Figure 10B illustrates the distribution of risk scores, survival status, and mRNA expression profiles. An increase in risk scores correlated with elevated patient mortality and decreased survival rates. Additionally, the heatmap demonstrated that the expression of these five molecules intensified alongside the augmentation of risk scores.

As depicted in Figure 10C, utilizing the timeROC package, the AUC values of the riskscore model fluctuated between 0.66 and 0.68, indicating consistent predictive performance of the riskscore model across different time points. Expression analysis (Figure 10D) showed that the expression levels of CDC20, ERCC6L, FAM136A, PLK1, and UBE2T were significantly higher in the high‐risk group than in the low‐risk group, suggesting a positive correlation between the overexpression of these molecules and increased risk, potentially contributing to the progression of LUAD.

## 4 Discussion

LUAD, a major subtype of non‐small cell lung cancer (NSCLC), undergoes a complex interplay of genetic and environmental factors in its initiation and progression. Mitochondria, serving as the “powerhouses” of cells, play a pivotal role in the development of various cancers, including LUAD, where their dysfunction or damage is crucial. Immune cells in the tumor microenvironment perform multifaceted roles, encompassing surveillance, elimination of tumor cells, and modulation of tumor growth. Notably, studies have revealed a close association between the enrichment of specific immune cell types and the abnormal expression of genes related to mitochondrial damage. This observation underscores the intricate relationship between mitochondrial health and the immune landscape within the tumor microenvironment, offering novel insights into the mechanisms underlying LUAD and potential therapeutic targets [9]. These mitochondrial damage‐related genes significantly impact the development of LUAD by modulating the metabolic pathways, proliferative capacity, apoptotic processes, as well as the invasive and metastatic potential of LUAD cells. This underscores the pivotal role of mitochondrial integrity in governing the tumorigenic progression, highlighting potential avenues for targeted therapeutic strategies [10, 11]. Specifically, abnormal expression of these genes can lead to mitochondrial dysfunction, which in turn fosters the malignant transformation and progression of tumor cells. For instance, mitochondrial dynamics, a process tightly regulated by fusion and fission proteins, exerts a direct influence on crucial cellular processes such as proliferation, energy metabolism, apoptosis, as well as cell invasion and migration. Thus, disruptions in these genes can significantly contribute to the aggressive behavior of LUAD [12, 13]. Furthermore, in LUAD, the activity of key proteins such as mitochondrial dynamin‐related protein 1 (Drp1) is intimately linked to the proliferation, invasion, and metastasis capabilities of tumor cells. This study conducted a thorough analysis to identify genes closely associated with mitochondrial damage in LUAD. Employing the SSGSEA method, we unveiled the significant infiltration of 10 distinct immune cell types in LUAD, including CD8 T cells, cytotoxic cells, eosinophils, immature dendritic cells (iDCs), macrophages, neutrophils, CD56‐low natural killer cells (NK CD56dim cells), natural killer cells (NK cells), plasmacytoid dendritic cells (pDCs), and effector memory T cells (Tem). These findings suggest a close correlation between the infiltration of immune cells and the abnormal expression of mitochondrial damage‐related genes. Specifically, the enrichment of CD8 T cells and NK cells, as the primary antitumor immune cells, may reflect the body's immune response against LUAD. These cells may be regulated by mitochondrial status in their activities of recognizing and killing tumor cells altered by mitochondrial damage genes. Cytotoxic cells, such as cytotoxic T lymphocytes and NK cells, directly kill tumor cells transformed by mitochondrial damage genes, and the enhancement of their number and function may indicate a better antitumor immune effect. The roles of macrophages and neutrophils in the tumor microenvironment are complex and diverse, as they can either promote tumor growth (M2‐type macrophages and N2‐type neutrophils) or inhibit tumor progression (M1‐type and N1‐type) [14, 15, 16]. Mitochondrial damage may affect the tumor‐related functions of these cells by altering their polarization states. Dendritic cells, as antigen‐presenting cells, are crucial in initiating and regulating immune responses [17]. Mitochondrial damage may impact their maturation, migration, and antigen‐presenting capabilities, thereby modulating the intensity of antitumor immune responses. The specific role of eosinophils in LUAD is not fully understood, but studies have suggested that they may play a part in antitumor immunity, and their enrichment may correlate with specific expression patterns of mitochondrial damage genes [18, 19]. Effector memory T cells are capable of rapidly responding to re‐exposed antigens, potentially playing a significant role in the recurrence or metastasis of LUAD. The findings of this study provide a theoretical reference for the development of tumor treatment strategies targeting mitochondrial dysfunction and immune microenvironment modulation. By regulating mitochondrial function and restoring normal metabolic pathways in tumor cells, it may be possible to inhibit their malignant transformation and progression. Furthermore, by enhancing the activity and function of antitumor immune cells, we can improve the body's immune response to tumor cells, potentially leading to more effective tumor treatments. Future research can further explore the specific molecular mechanisms between mitochondrial damage genes and immune cells, as well as the roles of these mechanisms in the initiation and progression of LUAD, providing a theoretical basis for the development of novel targeted therapies and immunotherapy strategies.

In addition, we conducted a GSEA on genes related to mitochondrial damage in LUAD. In our study, we conducted an immunoinfiltration analysis of mitochondrial damage‐related genes in LUAD, coupled with the construction of a classification and prognostic model integrated with weighted gene co‐expression network analysis (WGCNA) and machine learning algorithms. The results revealed a statistically significant enrichment of multiple gene sets that are pertinent to various biological processes and diseases. Notably, under the criteria of adjusted *p*‐value < 0.05 and FDR or *q*‐value < 0.25, we identified 82 significantly enriched gene sets, among which several are of particular interest and relevance to LUAD. One of the most striking findings is the enrichment of the gene set related to cellular senescence (REACTOME_CELLULAR_SENESCENCE). Cellular senescence is a state of irreversible growth arrest that can be triggered by various stressors, including mitochondrial damage. The significant enrichment of this gene set suggests that cellular senescence may play a crucial role in the development and progression of LUAD, potentially through mechanisms involving mitochondrial dysfunction. Furthermore, we observed the enrichment of gene sets involved in the formation of the β‐catenin/TCF transcription factor complex (REACTOME_FORMATION_OF_THE_BETA_CATENIN_TCF_TRANSACTIVATING_COMPLEX) and post‐translational protein modification (REACTOME_POST_TRANSLATIONAL_PROTEIN_MODIFICATION). These findings imply that alterations in transcriptional regulation and protein modification pathways may contribute to the oncogenic process in LUAD, potentially in response to mitochondrial damage. Additionally, the enrichment of gene sets related to histone acetylation (REACTOME_HATS_ACETYLATE_HISTONES) and deacetylation (REACTOME_HDACS_DEACETYLATE_HISTONES) suggests that chromatin remodeling and epigenetic regulation are also important in the context of LUAD. These processes may be involved in the regulation of gene expression in response to mitochondrial stress, thereby influencing tumor progression. Of note, we also identified the enrichment of gene sets associated with human cytomegalovirus (HCMV) life cycle events, including late events (REACTOME_HCMV_LATE_EVENTS), early events (REACTOME_HCMV_EARLY_EVENTS), and infection (REACTOME_HCMV_INFECTION). Although the direct link between HCMV and LUAD remains unclear, these findings raise the possibility that viral infections may contribute to the oncogenic process through interactions with mitochondrial damage‐related pathways. Lastly, the enrichment of the gene set related to systemic lupus erythematosus (KEGG_SYSTEMIC_LUPUS_ERYTHEMATOSUS) is intriguing, as it suggests potential shared mechanisms between autoimmune diseases and cancer. This finding may pave the way for future research exploring the role of autoimmune‐related pathways in LUAD development.

Subsequently, WGCNA and machine learning algorithms were employed to further screen genes, narrowing them down to the most crucial molecules, including 10 classification‐key molecules and 5 prognosis‐key molecules. Classification and prognosis models were constructed based on these molecules, respectively. Evaluation results of the classification and prognosis models demonstrated their outstanding performance, not only exhibiting good predictive ability within the internal dataset but also successfully validating the models' generalization capability in external independent GEO datasets, further confirming their stability and reliability. This also indicates that the key genes may play pivotal roles in the initiation and progression of LUAD related to mitochondrial damage.

It has been reported that due to the inhibition of PLK1 and mitochondrial dysfunction, the apoptosis rate of HCC cells increases, and a synergistic anti‐tumor effect can be observed both in vitro and in vivo [20]. While the aforementioned content primarily focuses on the behavior of PLK1 in HCC (hepatocellular carcinoma) and its relationship with mitochondrial dysfunction and apoptosis, this discovery also holds enlightening implications for understanding similar molecular mechanisms in LUAD. If a regulatory relationship between PLK1 and mitochondrial damage genes is confirmed, these genes will emerge as potential therapeutic targets. By jointly inhibiting PLK1 and these genes, it may be possible to more effectively inhibit LUAD cell proliferation and induce apoptosis. Further research into the interaction mechanisms of these genes is needed to provide novel strategies for LUAD treatment.

In addressing the research gaps and limitations, it is important to acknowledge that, despite the comprehensive bioinformatics analysis and machine learning techniques employed in this study, there are still several areas for improvement. Firstly, while we compared the strengths and weaknesses of various machine learning models and found the Random Forest (RF) model to be optimal for LUAD classification based on mitochondrial damage–related genes, the performance of these models may vary with different datasets and parameter settings. Therefore, further validation with larger and more diverse datasets is needed to confirm the robustness and generalizability of our findings. Secondly, although we constructed classification and prognosis models, the current study is limited to a bioinformatics analysis, lacking experimental validation. Future work should aim to validate the key genes and pathways identified through experimental approaches, such as in vitro and in vivo experiments, to further substantiate their roles in LUAD. Additionally, the study is constrained by the availability of public datasets, which may not fully represent the heterogeneity of LUAD. As such, there is a need to expand the sample size and include more diverse patient populations to enhance the generalizability of the models constructed.

## 5 Conclusion

This study focuses on the genes associated with mitochondrial damage in LUAD, revealing their crucial roles in immune infiltration, biological functions, signaling pathways, as well as disease classification and prognosis through bioinformatic analysis. The main findings encompass the following:

*Immune infiltration characteristics*: We identified immune cell types associated with mitochondrial damage, including CD8 T cells, cytotoxic cells, eosinophils, immature dendritic cells (iDCs), macrophages, neutrophils, CD56dim natural killer cells (NK CD56dim cells), natural killer cells (NK cells), plasmacytoid dendritic cells (pDCs), and effector memory T cells (Tem). These findings provide novel insights into the immune microenvironment of LUAD.

*Functional and pathway analysis*: We elucidated the biological functions of these genes in LUAD and the signaling pathways in which they participate offering a theoretical basis for the development of therapeutic strategies.

*Disease classification*: A classification model for LUAD based on mitochondrial damage‐related genes was constructed, demonstrating its exceptional classification performance.

*Prognostic assessment*: An effective prognostic model was developed, capable of predicting the survival outcomes of LUAD patients, thereby supporting individualized treatment approaches.

## Author Contributions

**Jirong Zhang:** conceptualization (equal), experimental design (lead), writing – original draft (equal), and writing – review and editing (equal). **Lin Lin:** conceptualization (equal), data collection and analysis (lead), writing – original draft (equal), and writing – review and editing (equal).

## Conflicts of Interest

The authors declare no conflicts of interest.

## Data Availability

The data used in this study are sourced from the public databases TCGA and GEO, which are publicly available. We confirm that other researchers can access these databases to obtain the same data as used in this study and conduct further analyses and research.
